# The Diagnosis and Medical Treatment of Intestinal Obstruction

**Published:** 1896-01

**Authors:** R. F. Williams

**Affiliations:** Richmond, Va.


### THE DIAGNOSIS AND MEDICAL TREATMENT OF INTESTINAL OBSTRUCTION.*

BY R. F. WILLIAMS, M.D.,
RICHMOND, VA.

*General Symptoms:*—In the *chronic* forms of intestinal obstruction, the first symptoms noticed is some disturbance of defecation. Usually, this is constipation, which has gradually increased, and which is often accompanied by tenesmus and pain. There may be diarrhea, with bloody and mucus stools, or constipation alternating with diarrhea produced by irritation of old fecal matter or passive hyperemia. If there be a change of form in the feces, they will usually be flat and ribbon-like; the appearance of pea-like lumps in the stool, like the feces of a goat, is not diagnostic of obstruction, as the same occurs in other forms of constipation. There is a gradually increasing meteorism. Peristaltic movements of the gut may be seen through the abdominal wall as this is thin from the general emaciation of the patient, and the gut wall is hypertrophied. Some authors have recommended this as a more or less efficient means of diagnosing the site of the obstruction; but, on account of the mobility of the gut and its frequent change of position, it is of doubtful value for this purpose.

Aortic pulsations may some times be felt transmitted through the distended gut.

Vomiting is a frequent symptom as the trouble advances, but it is rarely fecal.

Pain, colicky in character, is a usual symptom; although cases are recorded as proceeding without pain until complete occlusion. More or less anemia and emaciation are present, and may be severe in such cases caused by carcinoma and tuberculosis. The chronic form may follow on the subsidence of an acute attack; but it is more common for an acute attack to supervene in the course of the chronic trouble.

The symptoms of *acute* intestinal obstruction are sudden pain in

*Abstract of paper read before the Richmond Academy of Medicine and Surgery, October 22, 1895.

the abdomen, at first colicky, but becoming continuous, usually with exacerbations. The abdomen is tender and distended to a great or moderate degree according to the site of the obstruction; but there is no flatulence, and constipation is absolute except in intussusception, and in some instances, when fecal matter below the seat of obstruction may be voided.

Vomiting is a constant symptom, sometimes alternating with eructations. In the beginning of the trouble, the contents of the stomach are first vomited, then bile, and later, stercoraceous matter. This is not produced by a reversed peristalsis bringing the contents of the large gut into the stomach, but by putrid decomposition in the small gut as is shown by the fact that in obstruction of the small gut stercoraceous vomiting occurs, and is a more constant symptom in this form than in obstruction of the large gut. The vomiting is caused by stretching of the peritoneum and by irritation of the stomach, produced in the presence of abnormal matter which has entered when the pylorus is relaxed by pressure from within the gut.

There are rapidly developing symptoms of collapse. The pulse rapidly increases in frequency and becomes weak; the extremities cool and cyanotic; the eyes and cheeks sunken; the voice weak. The temperature is variable though it usually falls. The tongue is dry, and there is great thirst. The urine is scant and high colored, and may even be suppressed when the obstruction is high, due, probably, to the constant vomiting and consequent small amount of fluid absorbed. Respiration may be interfered with by the distention and pain. Peristaltic movements may be seen through the abdominal walls; but this is not a constant symptom on account of paresis of the gut wall, thick abdominal walls, etc.

The *diagnosis* of intestinal obstruction is always difficult and cannot be dogmatically given; as it must be made by differences in degree of symptoms for the most part; and, perhaps, more than in any other condition, is the judgment of the physician called in here.

Diagnosis as to the site of the obstruction. Abdominal inspection and palpation give indications of the location by the detection of well-defined masses or active peristalsis in the distended coils.

Rectal and vaginal examinations should always be made, as by these means, the condition of the pelvic organs can be discovered, and often, if the obstruction be low, it can be felt; while if high, the fingers will find the empty coils depending into the pelvis. Rectal examination with the whole hand is not to be recommended. Obstruction of the small gut gives rise to greater anuria, and there is, usually, an increased amount of indican in the urine due to the decomposition of albuminous matter, forming indal; while if the obstruction be in the large intestine, indican is not increased as the albuminous substances are not present there in such quantity.

In obstruction of the small gut there is greater vomiting, which becomes stercoraceous early, and less tympanites, the distention being in the upper part of the abdomen if the duodenum or jejunum is the site of the obstruction, and in the central part, if the ileum be involved. Prof. Monti, of Vienna, gives as a diagnostic point, pain beginning about the umbilicus and radiating toward the stomach when the duodenum or jejunum is the site; while, if the obstruction be lower down, pain begins in the cecal regions. He also considers the development of tympanites of diagnostic value, if the case be seen early enough.

In obstruction of the large gut, there is greater tympanites and vomiting comes on more slowly. Tenesmus and the passage of mucus and blood are commoner. For deciding the point of obstruction in the large intestine rectal examination will give evidence of the involvement of the rectum, and often, of the sigmoid flexure. Inflation with a bellows, or with bicarbonate of sodium and tartaric acid, is a useful means. Knowing the capacity of the large gut to be about six quarts, distention of the gut by the injection of known quantities of water will frequently aid in the diagnosis. For this purpose only a soft rubber tube should be used on account of the danger by a hard one. The patient should be anesthetized, and have his buttocks raised. In both of these last mentioned means care must be used, as cases are on record where such methods have caused ruptures. To further aid the diagnosis by this method, auscultation along the course of the large gut should be made, as the gurgling sound will give further evidence.

*Of the Nature of the Obstruction:—Hernia* should be excluded by a thorough examination of all possible points for its occurrence.

*Strangulation* is rare in the young. The history usually gives evidence of previous abdominal operations, peritonitis, or colic. The attack comes on suddenly, fecal vomiting occurs early; local tenderness is usually absent at first, but comes on later; constipation is absolute; fever may or may not be present. No tumor can be felt, as a rule.

*Intussusception* is commonly met with in children, occurring even in young infants. The onset is sudden. Pain, at first, is usually paroxysmal, and tenderness is often absent, pressure often giving relief. Fecal vomiting is not a common symptom. Constipation is not the rule, but if the invagination occurs in the small gut, it may exist, when there will usually be more hemorrhage. Usually, there are bloody stools, the amout of blood often reaching a severe degree of hemorrhage. There may even be diarrhea. Tenesmus is a frequent symptom, especially when the large gut is involved. If the affection be in the large gut, by rectal examination one may often touch the invaginated portion, feeling like a softened, patulous cervix. The presence of a sausage-shaped tumor can usually be found by abdominal palpation.

*Volvulus* occurs oftenest in men of middle and advanced life. The diagnosis is always difficult, and often laparotomy is the only means of making it. The usual sites are the lower part of the ileum, and, most frequently, the sigmoid flexure. The onset is sudden. Pain is, at first, intermittent, becoming constant later. Tympanites is marked, beginning frequently as a circumscribed area in the umbilical and epigastric regions, and tending to the left side. Constipation is absolute, and peritonitis appears early The passage of a soft rubber tube, and injection of water, is especially useful for diagnosis in this form of obstruction, though some object to it, saying it increases the trouble.

*Fecal impaction* occurs most frequently in women and lunatics. There is always a history of previous constipation, which, as a rule, gradually increases till the point of occlusion is reached; but the occlusion may be sudden. The commonest sites are the cecum and rectum. Constipation is usually absolute, but, when the impaction occurs in the rectum, the mass may be channelled, allowing the passage of feces from above, and the patient, for a long time, be ignorant of any trouble. There is no blood in such actions. Eructation is a common, and, often, distressing accompaniment of fecal impaction, but vomiting does not occur till late, and is very rarely stercoraceous. Pain and tympanites are also late symptoms. A tumor, which pits on pressure, can usually be felt by abdominal palpation, and if the rectum be the site, the trouble can be diagnosed by digital examination.

In impaction by gall stones, there is usually, a history of previous colics, and fecal vomiting and pain occur early. Jaundice is not often met with, and a tumor is rarely found. Constriction is diagnosed by a history of previous dysentery, syphilis, tuberculosis, typhoid, etc., in addition to the general symptoms of the chronic form of the trouble.

Obstruction due to paralysis of the gut wall from traumatism, or other cause, is usually accompanied by paralysis of other parts; and is diagnosed by the history of such paralysis.

The *course* of the affection is variable, in acute cases ending in a few hours, or extending over a week or two. About six weeks is the usual period. In chronic cases, the duration depends upon the cause. The prognosis is always grave, most cases ending fatally. In some cases there is a possibility of spontaneous recovery, as when in intussusception the gangrenous portion is passed. One reason, probably, of the great fatality, is the long delayed surgical treatment. Treatment for the relief of intestinal abstruction has been attempted in many ways—bullets in the sixteenth century, shot more recently; metallic mercury, purgatives, opiates, enemata, electricity, massage, and laparotomy. As the result of experience, it is now conceded that in most cases it is a waste of time to attempt alleviation by means of medical treatment, and that as soon as diagnosis has been made, operative treatment should be given. Purgatives should not be used, but in the early stages the administration of opium greatly relieves pain by checking peristalsis. It also lessens the vomiting, but it must be borne in mind that it obscures the symptoms. Enemata should be tried early, except, perhaps, in volvulus; although in most cases little good may be expected. When these have been used, and the water returns unchanged, the vomiting continuing, operation is indicated. Washing out the stomach is useful in relieving nausea and lessening the tension of the bowel. In some cases it has even proved curative.

For the relief of tympanites, puncture of the gut through the abdominal wall with a troca and canula, has been advised, but it is unsatisfactory, as, on account of the mobility of the gut, the portion below the obstruction may be punctured, and there may result a septic peritonitis from failure of the gut walls to close perfectly. For this purpose hot turpentine stupes may be tried, but are insufficient.

Mr. Jonathan Hutchinson in the *Report of the British Medical Association's* meeting in 1893, advocates taxis for acute intestinal obstruction, claiming that in most cases his results were excellent, whereas he knew of only one case cured by laparotomy. His method is, having anesthetized the patient, to invert, and then to forcibly push and shake the intestines from side to side, large enemeta being given while the patient is inverted. Then the patient is to be thoroughly shaken by four strong men, first in the inverted position, then in the erect. In some cases he saw no immediate results, but in one hour or two the bowels moved, and all went well.

In the acute affection, the food should not be administered by mouth, but only as nutrient enemata, otherwise it is immediately vomited and adds to the patient's discomfort.

Medical treatment is often efficacious and sufficient in two forms —intussusception and fecal impaction. Opium should be given in both cases, as it relieves the pain and vomiting, and in case of intussusception prevents further invagination. Enemata should be freely used. In intussusception, the patient should be put in the knee-chest position, and a soft rubber tube inserted, water being slowly injected, the nurse pressing the buttocks together to prevent its escape. In fecal impaction, continuous enemata for half an hour at a time should be employed. These may be of soap, water, oil, or turpentine and oil in the proportion of one ounce to the pint. Inflation with a bellows may be employed in cases of intussusception, instead of the injection of water, but care must be observed. The stomach should be washed in both cases, as it relieves vomiting and tension, and prevents the absorption of noxious matter. It has also proved curative.

For cases caused by paralysis, electricity and massage may be of benefit.

If relief is not speedily accomplished by measures indicated, operation should be performed.

